# Effect of Impregnated Mosquito Bed Nets on the Prevalence of Malaria among Pregnant Women in Foumban Subdivision, West Region of Cameroon

**DOI:** 10.1155/2020/7438317

**Published:** 2020-07-18

**Authors:** Ngouyamsa N. A. Sidiki, Vincent Khan Payne, Yamssi Cedric, Noumedem A. C. Nadia

**Affiliations:** ^1^Department of Animal Biology, Faculty of Science, University of Dschang, P.O. Box 067, Dschang, Cameroon; ^2^Department of Biomedical Sciences, Faculty of Health Sciences, University of Bamenda, P.O. Box 39 Bambili, Cameroon; ^3^Department of Microbiology, Haematology and Immunology Faculty of Medicine and Pharmaceutical Sciences, University of Dschang, P.O. Box 96, Dschang, Cameroon

## Abstract

**Background:**

Malaria is one of the major public health problems in many tropical developing countries including Cameroon. Impregnated mosquito bed nets are one of the control measures put in place by the WHO and adopted by the Cameroon's Ministry of Public Health to fight against malaria in pregnancy. This study was a population-based cross-sectional study that investigated the level of adherence, respondent's knowledge, altitude, and practices toward malaria prevention and control.

**Methods:**

To investigate this, a sample size of 410 pregnant women who were inhabitants of Foumban Subdivision was examined. Data on net ownership versus usage, pregnancy status, and socioeconomic background were collected using a questionnaire. Parasitological tests for malaria parasites were carried out using peripheral blood samples obtained from finger pricks of the pregnant women for the preparation of thick blood smear and RDTs.

**Results:**

Two hundred and eighteen tested positive (53.4%) with the highest prevalence occurring during the first trimester (79.6%) and in primigravidae (68.8%). Participants believed that mosquito bed nets can protect them against malaria infection. The highest number (81.0%) of the women who had mosquito nets acquired them during antenatal visits. Among those who possessed nets, 42.7% adhered to sleeping under them and few (50%) experienced problems of sweating, discomfort, and heat. Also, the study revealed a high prevalence rate of 63.8% for those who did not use nets during pregnancy as compared to those who owned and used them.

**Conclusion:**

The findings indicated that increased access to impregnated mosquito bed nets is required to lower the risk of malaria infection amongst pregnant women. The Cameroon government should improve health education to families within the locality and pursue an integrated approach to fight against mosquitoes during the rainy season.

## 1. Introduction

Malaria is a life-threatening chronic parasitic disease for nonimmune individuals and is spread by the bite of a female Anopheles mosquito, which results in the infection of red blood cells [1]. The vector is active between dusk and dawn, and the disease occurs mainly in tropical areas of Africa, Asia, and Latin America. It is the most dangerous parasitic disease in the world accounting for over 500 million clinical infections and 1 million deaths every year; conditions such as ecological change and economic and political instability, combined with escalating malaria drug resistance, have led to a worldwide regeneration of this parasitic disease [2]. The solemnity and gravity of this vector-borne disease occur in Sub-Saharan Africa, with over 90% of the world's malaria-related deaths occurring in this region and of the most malaria-endemic countries; Cameroon is ranked third in Central Africa and eleventh in Africa, [3] with a prevalence of 71 and 29 per 1000 in high and low transmission areas, respectively [4]. Malaria in pregnancy is an obstetric, social, and medical problem all over the world particularly in tropical and subtropical countries, and approximately twenty-five million pregnant women are currently at risk of malaria and according to WHO; malaria accounts for over 10,000 maternal and 200,000 neonatal deaths per year [4]. The percentage of the global population at risk has decreased from 77% at the end of the 20^th^ century to a low of 46% in 1994. It has been evaluated and estimated that by the end of the 21^st^ century, 48% of the global population will still be living and exposed to the risk of malaria infection [5].

In Cameroon, over 90% of Cameroon's population are at risk of infection by malaria [6] with about 6 to 8 mosquito bites per person per night [7]. About 41% of the population suffers at least one episode of malaria each year, with the highest mortality among pregnant women and under-fives [4]. Cameroon has characteristic typical African desert with both tropical and equatorial climates which favour the breeding of mosquitoes [8]. Cameroon's Ministry of Public Health reported that malaria causes 30 to 35% of overall mortality [9] and 67% of annual childhood mortality [10]. Transmission is endemic throughout the country, and this is mostly due to the increasing resistance of the parasite which in the greater majority is *Plasmodium falciparum* [5].

This disease can affect everyone, but individuals at high risk are children under five, pregnant women, people living under emergency situations, as well as immunosuppressed individuals. It has the main consequence in pregnancy being anaemia. Apart from anaemia, malaria infection can also lead to the accumulation of parasites in the placenta, foetal exposure, congenital infection, infant anaemia, infant morbidity and mortality, as well as maternal death [11]. It is due to these fatal consequences which could lead to death that malaria needs to be controlled during pregnancy and in other populations at high risk of infection. According to the WHO in 2003, the current prevention of malarial disease during pregnancy relies on three main strategies: providing pregnant women with insecticide-treated bed nets (ITNs), intermittent preventive treatment (IPT) with antimalaria medication, and case management [12]. Protecting the bed with long-lasting insecticide-treated nets prevent bites from malaria-infected mosquitoes and kill the vectors. Sleeping under ITNs was demonstrated to reduce the risk of a pregnant woman being infected with malaria thus lowering the risk of maternal related anaemia and low birth weight. It has also been demonstrated that pregnant women who use insecticide-treated nets effectively during pregnancy are at low risk of infection and lastly the use of ITNs has reduced malaria burden worldwide [13]. Malaria is a huge socioeconomic problem in tropical countries. Its treatment and control requires much money, both for individuals and for the government. The socioeconomic consequences at the personal level are numerous and result in reduced productivity as a whole. A striking correlation has been shown between malaria and poverty [14].

Given that Foumban Subdivision falls in the area of stable transmission in Cameroon where malaria infection is asymptomatic and also the majority of these pregnant women when pregnant start antenatal consultation late or not at all and this predisposes them to malaria infection. Taking into consideration the methods of control put in place by the WHO, this study investigated the impact of impregnated mosquito bed nets on the prevalence of malaria among pregnant women in the Foumban Subdivision, West Region of Cameroon.

## 2. Material and Methods

### 2.1. Study Area

The study was conducted in Foumban Subdivision; headquarters of Noun Division which is the largest Division in the West Region of Cameroon. It is a city found in the North East of Bafoussam. Foumban Subdivision is located 5°43′-5°43′ north latitude and 10°53′-10°55′ east longitude and is spread on 7000 ha on 900-1200 m of altitude on average.

### 2.2. Study Design

This was a population-based cross-sectional study using the convenience sampling method in which pregnant women in their households were recruited until a total number of sample size was achieved. During the course of this study, pregnant women who were living in Foumban Subdivision were recruited as participants. A sample size of 410 was calculated based on a 2-sided hypothesis tests using Epi-info with 80% power, confidence interval of 95%, expected frequency for ITNs coverage of 50%, and a precision of 10%, and has added additional 10% to account for nonresponse.

### 2.3. Inclusion and Exclusion Criteria

#### 2.3.1. Inclusion Criteria

Pregnant women who signed a free informed consent and are permanent members of the city were included in the study. Also, only pregnant women with good health status of different age groups were included in the study.

#### 2.3.2. Exclusion Criteria

For this study, pregnant women who were mentally handicapped or taking antimalarial therapy or who had been treated with antimalaria medications or drugs during the period of study or refused to sign the informed consent were excluded. In addition, it was also excluded those who were not resident in the studied locality or study area.

### 2.4. Study Population

Four hundred and ten participants were randomly recruited in their households from April to July 2018 to participate in this study. Pregnant women of all trimesters, parity, and age group with a mean age of 26.52 ± 7.17 were involved in the study. The majority of the participants were Muslims and the Bamouns were in the majority. Three educational levels were represented, i.e., Primary, Secondary, and Diploma.

### 2.5. Data Collection

Pregnant women in their first, second and subsequent pregnancies, different age groups, and different trimesters were recruited in the study after they signed an informed consent form. Data were collected using a standardised questionnaire, RDTs, and prepared thick blood samples for plasmodium detection. A coded number was assigned to participants that were recorded on the questionnaires, the prepared slides, and RDTs.

### 2.6. Parasitological Examination

Blood samples were collected from a finger prick after cleaning it with cotton wool soaked in alcohol by the principal investigator. This blood was then used to perform the Rapid Diagnostic Test (RDTs) and thick blood smear. Concerning RDTs, the First Response® Malaria Ag. P. *falciparum* (HRP2) Card Test was used according to the rules of Clinical and Laboratory Standard Institute for the identification of malaria parasites. Regarding the preparation of thick blood smear, a large drop of blood was placed on a microscopic slide and using the edge of another clean slide spread the blood in a circular manner with 6-15 movements to make an area of 1 cm^2^. The prepared slides were then air-dried in a horizontal position and placed in the slide box and carefully transported to Foumban District Hospital for parasitological examination. Once in the laboratory, the slides were stained using a 10% Giemsa solution and observed under a light microscope at objective ×100 after adding oil immersion for the identification of malaria parasites.

### 2.7. Statistical Analysis

The data obtained was entered and stored into the computer using MS excel software 2007 and was analyzed using SPSS version 20 software. For categorical or qualitative variables, data were presented as frequencies, while continuous variable (age) data were presented as means. A chi-square test was used for comparisons between respondent characteristics and malaria prevalence at 0.05 significant levels. A univariate analysis was also carried out to independently determine whether any of the prospectively defined independent factors (age, net usage, and sleeping behaviour) was significantly associated with malaria prevalence. To assess relative risk, odds ratio at 95% confidence interval was computed using Medcalc software.

## 3. Results

### 3.1. Sociodemographic Characteristic of the Studied Population

Among the 410 pregnant women who participated in the study, the majority of them were urban residents, Bamouns, and Muslims. About 84.4% of the respondents were married, and most of them were housewives during data collection. Their ages ranged between 14 and 47 years with a mean age of 26.52 ± 7.17. Pregnant women aged between 14-24 years were more represented with 44.9% compared to others. Furthermore, most of the respondents, 180 (43.9%) were housewives, 144 (35.1%) were holding a small business, 32 (7.8%) were student, 26 (6.3%) had no job, 24 (5.9%) were civil servant, and 4 (1.0%) were farmers. About 266 (69.8%) had a secondary level of education, 104 (25.4%) primary education, 3.4% (14) had a higher level of education or at least a diploma, and 6 (1.5%) had no formal education. Regarding the ethnic group of the study population, 344 (88.8%) were Bamoun, 22 (5.4%) Bamelike, 12 (2.9%) Haoussa, 10 (2.4%) were from other tribes, and 2 (0.5%) Borroro.

### 3.2. Knowledge and Perception of Mosquito Net as Malaria Prevention Method in Pregnant Women Not Having Mosquito Net

A total number of 202 respondents who did not have mosquito bed net had good knowledge of malaria transmission. Of this total number examined, 67.3% (144) knew that malaria is transmitted through mosquito bites. Respondents also declared that malaria is transmitted through water 18.7% (40) and mangoes 5.6% (12). Others mentioned that they do not know the cause of malaria 8.4% (18). The main reasons of not having mosquito bed nets were financial problems and availability during the period of free mass distribution. At least nine over ten (89.6%) of the respondents declared that if they are given mosquito bed nets they shall use it effectively. Few but not negligible proportion of respondents (10.4%) declared that they could not use ITNs if given to them despite all declared that it is to protect against malaria. Regarding other methods of prevention mentioned by the respondents, use the IRS of insecticide, destruction of breeding sites, the use of insecticide bomb accounted for 1.9% (4) each. Only two respondents declared clearing bushes to fight against transmission while more than 95% were using nothing to prevent malaria as shown in Table 1.

### 3.3. Net Ownership and Usage

Of 410 respondents, 50.7% (208) did not have mosquito bed net and 49.3% (202) had a mosquito net. The study recorded 81.0% (162) of the respondents who had acquired their net during the antenatal visit, 16.5% (33) from Ministry of public health during free distribution, 2.0% (4) purchased theirs from the supermarket, and 0.5% (1) had from gift. Looking at the net usage, 42.7% (70) used their net and 57.3% (94) did not use theirs during pregnancy. The study also measured the frequency of net usage per week and revealed that 13.4% (18) used one to two times per week, 16.4% (22) used 3 to 5 times a week, 68.7% (92) used more than 5 times a week, and 1.4% (2) had never used theirs. Moreover, we equally examined net usage during the year, and 54.7% (82) said they used their net throughout the year, 44.0% (66) said they used mostly during the rainy season, and 1.3% (2) said is during the dry season. Among these respondents that possessed nets, 58.0% (94) tucked into the mosquito bed net regularly; however, 25.0% (42) tucked in once a while 16.0% (26) had never tuck in. This result is highlighted in Table 2 below.

### 3.4. Shape of Mosquito Bed Net Preferred by Pregnant Women

**Figure 1:**
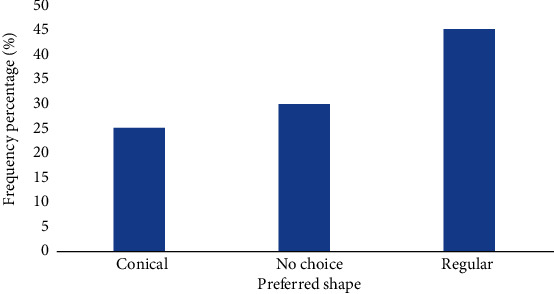
Preferred shape of mosquito bed net.

Out of 202 participants that had mosquito bed nets, 45% loved using bed nets which have a regular shape, 30% had no choice, and about 25% preferred conically shaped of net as seen in Figure 1 below.

### 3.5. Problems Encountered during Bed Net Usage

**Figure 2:**
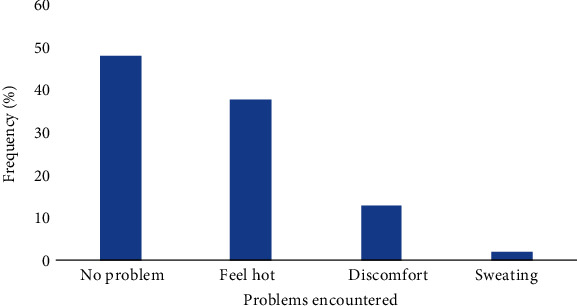
Problem encountered during the use ITNs.

A large portion of respondents, 48% had no problem or challenge during the use of a mosquito bed net. However, 38% of them mentioned that they feel hot when sleeping under mosquito bed nets, while 13% experienced had discomfort and 2% sweated during net usage. This best demonstrated in Figure 2.

### 3.6. Reason for Sleeping under Mosquito Bed Net

The respondents who owned mosquito nets indicated several reasons why they were sleeping under them, among which 140 (88.61%) claimed to sleep under the mosquito net in order to protect themselves, 16 (10.13%) were protecting themselves and children, 2 (1.3%) were protecting their children, and 2 (1.3%) were sleeping under the net to avoid malaria. This is well presented in Table 3.

### 3.7. Other Control Measures Used by Pregnant Women Having Nets

**Figure 3:**
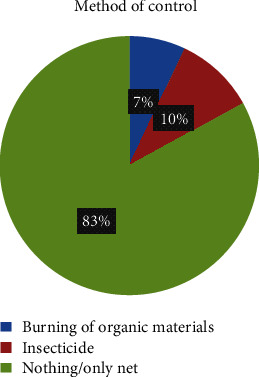
Other control measures used by pregnant women having nets.

Over 83% of those who were having mosquito bed nets relied on it to fight against malaria. However, 10% used insecticide as another control measure and 7% burnt organic materials. This is presented in Figure 3.

### 3.8. Overall Prevalence of Malaria among Pregnant Women

Among the 410 pregnant women examined, a total of 218 (53.4%) were found to be positive for malaria parasites. The overall prevalence of malaria among pregnant women was 53.4% as presented in Table 4.

### 3.9. Bed Net Usage and Risk of Malaria Infection

The study observed that 50.7% of the respondents owned nets. Concerning net usage, the rate of infection was 40.0% for those who owned and used mosquito bed nets and 63.8% for those who owned them but did not use it during pregnancy. There was a significant difference between the net usage of net and malaria parasitemia (*P* < 0.005). However, the risk was high for those who did not use the net during pregnancy (Table 5).

### 3.10. Sleeping Behavior and Risk of Malaria Infection

It revealed that those who slept late were highly infected with a prevalence rate of 65.2% as compared to those who slept early with prevalence of 48.0%. Those who go to bed late (after 10 pm) had two times the risk of being infected with malaria as compared to those who go to bed early (before 10 pm). There was a significant difference between the two studied parameters.

These findings also revealed that 54% for those who wake up early (before 6 am) and the trend observed for those who wake up late was slightly lower (51.4%). Also, no significant difference was recorded between the studied parameters (*P* > 0.05) and both those who wake up early and those wake up late were at the same risk of contracting malaria. This is well demonstrated in Table 6.

### 3.11. Educational Level, Age Group, and Malaria Infection

In this study, 100% of those who had no formal education were infected, 53.8% of those with primary education, 51.4% of those with secondary education, and 71.4% of those who had acquired postsecondary and post-high school education had positive parasitemia (Table 7). The relative risk was high for those who had no formal education, and there was no significant difference observed between the studied parameters (*P* = 0.05).

One hundred and eighty-two respondents aged between 14-24 years were examined; 106 (58.2%) were positive for malaria and had the highest prevalence. There was a significant difference between individuals having 34 years and above and other classes of age (*P* value < 0.05). The risk factor was high for pregnant women belonging to 14-24 and 25-34 years as seen in Table 7.

### 3.12. Parity, Gestation Period, and Risk of Malaria Infection

The highest infection rate of 68.8% was recorded for primigravidae followed by secundigravidae at 52.9% while the lowest (47.5%) was recorded in multigravidae (Table 8). The primigravidae were most likely to be tested positive for malaria infection. Respondents in their first trimester registered the highest infection rate of 79.6%.and had the highest risk of being infected with malaria as compared to others.

## 4. Discussion

A considerable percentage of the respondents were aged between 14-24 years, and majority of them were married (84.4%). About 44% of pregnant were housewives (43.9%), Bamoun 88.8%, and Muslims (87.3%). Those with secondary level education constituted the highest percentage (69.8%).

Approximately all the respondents who had mosquito bed nets had good knowledge of the cause and symptoms of malaria 202 (49.3%). Among those pregnant women who did not have net, 144 (67.3%) knew that it was transmitted by mosquito bites and some associated malaria to contaminated water, mangoes, and others do not know the main cause of malaria. This finding corroborates with that of Tassew et al. who demonstrated that the majority of the respondents who participated in his study knew that malaria is transmitted through mosquitoes even though the least number did not know what causes malaria [15].

The study examined bed nets ownership by pregnant women in Foumban Subdivision. Results revealed that over 202 (49.3%) of the participants owned net during pregnancy. This finding corroborated [16] who reported 47% of net ownership among households. This is far less than the coverage of 80% reported in Obala, Cameroon in 2009 [17].This difference may be due to the fact that the study in Obala was conducted two years after the distribution campaign and some people still had their net intact. The majority of the respondents were in agreement that nets protected them against mosquito bites. This is unlike the findings by Njoroge et al. who reported that nets do not protect against malaria infection, but instead people who have grown up without using nets avoid using them even during pregnancy [18]. The results showed that majority of MBNs were obtained from ANC and during the immunization campaign. Only 4 (2%) of the MBNs found among pregnant women were purchased from the market. A similar situation was reported in Nigeria by Singh and Singh who reported the highest place of acquisition of MBNs by pregnant women was at ANC [19]. The utilization of MBNs by pregnant women lagged behind ownership that is 42.7% (70) of those who owned MBNs used them during pregnancy. This corroborates with the findings of Afolabi et al. who reported high possessions of MBNs but low utilization in Nigeria [20]. This implies that a great proportion of the vulnerable groups were not protected against malaria with MBNs and this potentially placed them at higher risk due to mosquito “diversion effect”. This corroborates the report made by Park [21]. This low utilization of MBNs implies that the burden of malaria morbidity and mortality among vulnerable groups (Pregnant women) in Foumban Subdivision will continue to persist and even increase if nothing is done to increase net ownership and utilization during pregnancy. The majority of the respondents, 44% indicated that they use their net throughout the year, 54.7% mentioned the rainy season as the main period of usage, and 1.3% mention the dry season as the main period. Kimbi had also reported that using MBNs during the period of the rainy season coincided with the peak of malaria transmission [16]. The rectangular bed net was preferred (45%) by most of the respondents because it is more spacious while the conical net is easier to mount and more convenient for baby's cot and for small rooms. Therefore, shape preference of MBNs remains very subjective and depends to a large extent on the individuals concerned. Ngang'a et al. also reported similar results [22]. Also, respondents cited feeling hot, sweating, and discomfort as the main hindrance to proper net usage of net especially during the dry season. This is also similar to the study conducted by Njoroge et al. who reported that such problems where respondents claimed that they felt hot under a mosquito bed net and therefore uncomfortable especially during the dry season [18]. Furthermore, he reported that sleeping on the floor or outside made it difficult to use MBNs especially during hot seasons. Most of the respondents tuck in their MBNs before they slept, and this is in accordance with the study conducted by Ngang'a et al. who reported that most of his respondents tuck into nets before sleeping and that the use of MBNs reduced malaria infection [22].

Furthermore, 93.3% relied on MBNs to fight against malaria and a few number mentioned other preventive measures such as IRS, insecticide bomb, destruction of breeding sites with 1.9% each, and clearing bushes with 1%. This finding is similar to Tassew et al. in Ethiopia who reported that 87% of the household depend on MBNs to fight against malaria. Also, those who were not having MBNs mentioned that if they give they will use it effectively and they also indicated that they relied on MBNs to fight against malaria. More than 67.3% (144) of the studied participants who did not have mosquito bed nets mentioned mosquito bites as the main cause of malaria [15]. This is in accordance pursued in Africa where mosquitoes were identified as a cause of malaria by people with better formal education where mosquitoes were identified as a cause of malaria by people with better formal education [23].

Respondents who did not have mosquito bed nets were in agreement that nets can protect them against malaria infection. This result is in agreement with the findings of Njoroge et al. who reported that net protects against malaria infection [18]. They also cited several reasons why they do not have mosquito bed nets including; nonavailability during the period of distribution, financial problem, and the stock finished during distribution and were not yet given. Similar findings are reported by Kimbi who stated several reasons why pregnant did not possess net and also proposed a 100% subsidy by the government [16]. These respondents also mentioned that if they are given net they will use it effectively to protect their selves, and they relied most on mosquito nets to protect themselves against malaria (93.3%). The least number stated other control measures such as clearing of bushes, IRS, destruction of breeding sites, and insecticide bomb. This is similar to the study of Njoroge et al. who stated that the majority of pregnant women relied on nets to protect themselves against malaria infection, and other control measures such as insecticide, clearing bushes, and destruction of breeding sites were least used to control malaria [18].

The study highlighted that pregnant women with low income levels were highly infected with malaria (66.7%) as compared to those with high and average income levels. This finding agrees with [24, 25] who demonstrated that malaria infection is known to be an infection of poverty and also a cause of poverty and people with low income levels at high risk of infection.

The highest parasitaemia was reported in respondents who went to bed late (after 10 pm). The time of going to sleep may determine the likelihood of being bitten by mosquitoes, hence infection by malaria. People who stay awake for a long time probably expose themselves to mosquito bites, whereas those who sleep early cover themselves hence reducing the likelihood of the chance of being bitten. This is similar to what was obtained in a study carried out in Kenya by Mukanda [26]. They reported that pregnant women who went to bed late (8 pm-10 pm) where highly exposed and had a high prevalence compared to those who went to bed early.

This study also revealed that the highest parasitemia was observed among respondents who wake up early from the bed as compared to those who wake up late. Those who wake early had the highest risk of being infected. These findings have not been confirmed in other studies and need further investigation, though Kline [27] suggested that mosquitoes become active at night.

Women who owned mosquito bed nets and used them had less malaria infection as compared to those who had nets but did not use them; hence, there was a significant difference in malaria parasitemia between those who did not use and those who used net during pregnancy. Also, those who had nets but did not use them were at higher risk of being infected. Bed net usage during pregnancy therefore reduces cases of malaria. This finding corroborates with what was reported in Western Kenya by Hawley et al. [28]. This finding is also similar to that of Tokponnon et al. [29] where they demonstrated the impact of LLITNs on children less than five years in reducing malaria infection. Respondents were found to possess LLITNs of three brands (olyset, interceptor, and permanent); from this study, we observed that these different brands had an effect on the prevalence of malaria.

The overall prevalence of malaria infection observed in this study was 53.4%. This finding is comparable to other works carried out in other malaria-endemic areas; 57.5% reported in Gabon by Bayo-Akotet et al. [30], 54% in South East Nigeria by Nduka et al. [31], and 52% in a Suburb of Lagos Nigeria by Raimi and Kanu [32]. It was much lower than the study conducted among Cameroonian pregnant women by Walker et al. [33] who reported an overall prevalence of 82.4% and 65% observed in Nigeria by Maureen et al. [34]. This designation was much higher than that observed in other studies; 42% in Ghana by Mockenhaupt and his collaborators, 26% reported in Nigeria by Michael et al. [35], 26.7% reported in Malawi [36] and 41% observed in Uyo [37]. The high malaria prevalence observed in this study may be due to environmental conditions such as sanitation problems (poor drainage/sewage disposal systems) and flooding which are suitable breeding habitats for malaria vectors. The high prevalence reported in this study may also be due to multiple factors including seasonal changes, the intensity of the transmission, and characteristic of the study population (knowledge on what causes malaria and preventive measures against mosquito bites, parity, and HIV status). It may also be due to the Human chorionic gonadotropin and prolactin which suppresses the immune response of pregnant women, therefore making them susceptible or vulnerable to malaria infection compared to nonpregnant women.

Eighty-six (79.6%) women in their first trimester were highly infected than those in their second and third trimester with prevalence 46.6% (68) and 41.6% (64), respectively. The highest prevalence was obtained during the first trimester of pregnancy and decreased steadily during the second and third trimesters. This finding agrees with reports of other studies in which the peak prevalence was in weeks 10-20 of pregnancy [35, 38]. This high prevalence may be due to the expression of adherent proteins on the surface of infected red blood cells (IRBCs), enabling the IRBCs to adhere to microvascular capillaries of vital organs causing severe parasitological conditions [39]. In addition, it may also be due to the fact that pregnant women usually do not attend antenatal visits early in pregnancy and a large proportion of them might have unrecognized and untreated malaria infection as most infections are asymptomatic [40], another reason could be because of peak prevalence of *P. falciparum* infection occurring between the 9 and 16 weeks of gestation [13]. In contrast, other findings observed that the prevalence of infection was higher during the second trimester of pregnancy [34]. Amira reported the highest prevalence during the third trimester in the study conducted in Sudan [25].

The current study equally revealed that the prevalence of malaria infection is more in primigravidae 66 (68.8%), followed by secundigravidae 36 (52.9%), while multigravidae had only 116 (47.5%). This finding is supported by the observation from other investigations, where primigravidae was reported to be highly susceptible to malaria infection [32]. It is also similar to that reported in Malawi by [36] who reported a prevalence of 64%. However, this finding disagrees with that of [35] who had a prevalence of 26%. Primigravidae have been reported to be at greatest risk of malaria in pregnancy because of a lack of specific immunity to malaria which is acquired from exposure to malaria parasites during pregnancy [40]. This high prevalence of malaria infection observed among primigravidae may also be due to a substantial reduction in the level of immunity associated with first and second pregnancies. At higher parities, there appear to be a boosting of immunity with successive pregnancies provided there is exposure to the malaria parasite. In areas of stable malaria transmission in Sub-Saharan Africa, primigravidae and to a lesser extent secundigravidae are at higher risk of malaria infection and low birth weight [41].

Malaria parasitemia was more prevalent in women aged between 14-24 years (58.2%) and 25-34 years (53.2%) but was least among women from 34 years and above (41.7%). Malaria parasitaemia in relation to age showed that young pregnant women had the highest malaria prevalence rates. This finding is similar to that reported in Nigeria [31, 42]. The different rates of infection observed among these age groups could be attributed to the level of acquired immunity that increases with age, which may also be associated with protection from malaria infection. In contrast, other studies reported a higher prevalence in older pregnant women (86.2%) from age greater than 34 years; older pregnant women had a significant association with the malaria parasite [34]. According to his findings, malaria infection is high in younger women but reduces steadily to reach older women and younger women who were at high risk of infection. This is similar with the results obtained from other studies [35] in Nigeria.

## 5. Conclusion

Malaria is still a major public health problem among pregnant women in Foumban Subdivision. Increase efforts should be made in order to enhance sensitization and knowledge on the importance of using ITNs during pregnancy. Furthermore, health education and other methods of malaria prevention should be made available to pregnant women as they attend antenatal clinics irrespective of the trimester of pregnancy.

## Figures and Tables

**Table 1:** Knowledge and perception on mosquito net as malaria prevention method of pregnant women not having mosquito net in Foumban.

Indicator	*N*	%
Method of transmission		
Unknown	18	8.4
Mango	12	5.6
Mosquito bites	144	67.3
Water	40	18.7
Use of net effectively if received one		
No	22	10.4
Yes	190	89.6
Do you believe net can protect you?		
Yes	210	100
No	0	0
Reason for not having net		
Do not like	22	10.5
Financial problem	4	1.9
It was finish	2	1
Not available during distribution	126	60
Not yet given	56	26.7
Malaria prevention tools		
Cleaning bush	2	1
Destruction of breeding site	4	1.9
IRS	4	1.9
Insecticide bomb	4	1.9
Nothing	196	93.3

**Table 2:** Net ownership and level of adherence to the mosquito bed net.

Indicator	*N*	%
Possession of mosquito net		
No	208	50.7
Yes	202	49.3
Net brand		
Interceptor	50	25.0
Olyset	100	50.0
Permanet	50	25.0
Source of net		
Antenatal visit (hospital)	162	81.0
MoPH (free distribution)	33	16.5
Gift	1	0.5
Purchased	4	2.0
Net usage		
Yes	70	42.7
No	94	57.3
Net use per week		
1 to 2	18	13.4
3 to 5	22	16.4
>5	92	68.7
Never	2	1.4
Period of net usage		
Dry season	2	1.3
Rainy season	66	44.0
Throughout the year	82	54.7
How frequent do you tuck in		
Everyday	94	58.0
Never use	26	16.0
Once a while	42	25.0

**Table 3:** Reasons for sleeping under the net.

Indicator	*N*	%
Avoid malaria	2	1.3
Protect myself	140	88.61
Protect child	2	1.3
Protect myself and child	16	10.13

**Table 4:** Prevalence of malaria among pregnant women Foumban.

Parasitemia (%)	*N*° of individuals studied	Percentage
Positive	218	53.4
Negative	192	46.6
Total	410	100

**Table 5:** Net usage and risk of malaria infection.

Usage of net	Prevalence (%) *n*/*N*	OR (95% CI)	*P* value
No	(63.8) 60/94	1^b^	NA
Yes	(40.0) 28/70	0.22 (0.12-0.39)	0.002

^∗^(%) *n*/*N*: (percentage) number tested positive/number tested; OR (95% CI); ^∗∗^NA: not applicable; 1^b^: reference category.

**Table 6:** Sleeping behavior and risk malaria infection.

Characteristic	Prevalence (%) *n*/*N*	OR (95% CI)	*P* value
Time of going to bed			
Sleeping early (before 10 pm)	(48.0) 72/150	1^b^	NA
Sleeping late (after 10 pm)	(65.2) 30/46	2.03 (1.02-4.03)	0.04
Time of waking up			
Waking up late(after 6 am)	(51.4) 76/148	1^b^	NA
Waking up early (before 6 am)	(54.2) 26/48	0.81 (0.46-1.71)	0.73

^∗^(%) *n*/*N*: (percentage) number tested positive/number tested; OR (95% CI); ^∗∗^NA: not applicable; 1^b^: reference category.

**Table 7:** Educational level, age group, and malaria infection.

Characteristic	Prevalence (%) *n*/*N*	OR (95% CI)	*P* value
Level of education			
None	(100) 6/6	1^b^	NA
Primary	(53.8) 56/104	0.08 (0.0-1.6)	0.1
Secondary	(51.4) 146/284	0.08 (0.0-1.46)	0.08
Age			
14-24 year	(58.2) 106/182	1^b^	NA
25-34 year	(53.2) 82/154	0.82 (0.53-1.26)	0.35
>34 year	(41.7) 30/72	0.51 (0.29-0.89)	0.02

^∗^(%) *n*/*N*: (percentage) number tested positive/number tested; OR (95% CI); ^∗∗^NA: not applicable; 1^b^: reference category.

**Table 8:** Parity, gestation period, and risk of malaria infection.

Characteristic	Prevalence (%) n/N	OR (95% CI)	*P* value
Parity			
Primigravidae	(68.8) 66/96	2.42 (1.47-4.0)	<0.001
Secundigravidae	(52.9) 36/68	1.24 (0.72-2.13)	0.43
Multigravidae	(47.5) 116/244	1^b^	NA
Trimester			
First	(79.6) 86/108	1^b^	NA
Second	(46.6) 68/146	0.22 (0.12-0.39)	<0.001
Third	`(41.6) 64/154	0.18 (0.10-0.32)	<0.001

^∗^(%) *n*/*N*: (percentage) number tested positive/number tested; OR (95% CI); ^∗∗^NA: not applicable; 1^b^: reference category.

## Data Availability

Data and material are available to other researchers upon request.

## Abbreviation

WHO: World Health Organisation
MBNs: Mosquito bed net
IPT: Intermittent preventive treatment
RDTs: Rapid diagnostic tests
SPSS: Statistical package for social science
ITNs: Insecticide-treated nets
CI: Confidence interval
OR: Odds ratio.
